# Real‐world results with IgPro20 for hypo‐ or agammaglobulinemia in Japan

**DOI:** 10.1111/ped.15362

**Published:** 2022-11-27

**Authors:** Kohsuke Imai, Tomonori Ishii, Shigeaki Nonoyama, Takahiro Yasumi, Hirokazu Kanegane, Taku Fukushima, Masayuki Matsumaru, Tetsushi Akasaki, Hideo Usui

**Affiliations:** ^1^ Department of Community Pediatrics, Perinatal and Maternal Medicine Tokyo Medical and Dental University (TMDU) Tokyo Japan; ^2^ Clinical Research, Innovation and Education Center Tohoku University Hospital Miyagi Japan; ^3^ Department of Pediatrics National Defense Medical College Saitama Japan; ^4^ Department of Pediatrics Kyoto University Graduate School of Medicine Kyoto Japan; ^5^ Department of Child Health and Development Tokyo Medical and Dental University (TMDU) Tokyo Japan; ^6^ Medical Affairs CSL Behring K.K. Tokyo Japan; ^7^ Department of Pharmacovigilance CSL Behring K.K. Tokyo Japan

**Keywords:** agammaglobulinemia, hypogammaglobulinemia, immunoglobulins, postmarketing product surveillance, subcutaneous injections

## Abstract

**Background:**

Subcutaneous immunoglobulin is one of the standard treatments for hypogammaglobulinemia in primary immunodeficiencies (PID) worldwide. In Japan, IgPro20 (Hizentra^®^; l‐proline‐stabilized 20% human subcutaneous immunoglobulin) is approved for agammaglobulinemia or hypogammaglobulinemia due to PID or secondary immunodeficiency (SID); however, its safety and effectiveness has not previously been assessed in a real‐world setting.

**Methods:**

This multicenter, open label post‐marketing surveillance study was conducted between January 2014 and March 2019. Patients who received IgPro20 due to PID or SID were included after informed consent. Physicians completed a case report form for each patient. Safety was determined from reported adverse events (AEs), adverse drug reactions, and serious AEs (SAEs); effectiveness was assessed by infection rates after the first IgPro20 dose.

**Results:**

Of 85 patients receiving IgPro20 in the safety analysis, 39 developed AEs (45.9%; PID *n* = 28, SID *n* = 11). At least one adverse drug reaction was observed in 27 patients (31.8%; PID *n* = 21, SID *n* = 6), and the most common were injection site reactions (*n* = 17, 20.0%). Four patients (PID *n* = 3, SID *n* = 1) reported SAEs but two were unrelated to IgPro20 administration. The infection rate decreased from 0.54 per patient during the 6 months before IgPro20 to 0.39 per patient during IgPro20 treatment. Serious bacterial infections occurred in six patients before IgPro20 (7.9%; PID *n* = 2; SID *n* = 4) but in only one patient with SID during IgPro20 treatment (1.2%).

**Conclusions:**

In Japan, IgPro20 was considered safe and effective among patients with agammaglobulinemia or hypogammaglobulinemia due to PID or SID.

## INTRODUCTION

Primary immunodeficiencies (PIDs) or inborn errors of immunity (IEI) are a group of more than 430 inherited diseases in which patients have an increased incidence and severity of infections, immune abnormalities with autoimmune diseases and abnormal inflammatory responses, and a predisposition to malignancy.^1^ The most common types of PIDs/IEIs are associated with a deficiency in antibody production, including common variable immunodeficiency (CVID) and X‐linked agammaglobulinemia (XLA).^2^, ^3^ Agammaglobulinemia or hypogammaglobulinemia may also develop in patients with secondary immunodeficiencies (SID), either as a result of drug‐induced adverse effects from various treatments, such as immunosuppressive agents, steroids, radiation therapy, monoclonal antibodies, kinase inhibitors, or organ transplantation, or due to protein‐losing gastroenteropathy (PLGE), nephropathy, or hematologic malignancies, such as chronic lymphocytic leukemia, lymphoma, and multiple myeloma (MM).^4^ Aside from antibiotics, the standard of care for patients with agammaglobulinemia or hypogammaglobulinemia due to PID or SID is immunoglobulin G (IgG) replacement therapy, which aims to reduce the risk and severity of infections and prevent a long‐term decline in organ function. Immunoglobulin G can be administered as intravenous IgG (IVIG) or subcutaneous IgG (SCIG). Intravenous IgG first became available during the 1980s and SCIG during the 2000s.^5^, ^6^ Although IVIG and SCIG have similar effectiveness in reducing infection rates, SCIG preparations provide more stable blood levels of IgG and are associated with fewer systemic adverse effects.^7^ SCIG can also be self‐administered by patients or care‐givers at home, increasing treatment convenience for patients, who do not need to attend IgG infusion clinics as often.^8^

IgPro20 (Hizentra^®^, CSL Behring K.K.) is an l‐proline‐stabilized, 20% liquid preparation of pH 4‐treated acidic human immunoglobulin for subcutaneous injection. In phase 3 clinical trials in patients with PID from Japan, the USA, and Europe, IgPro20 provided sustained serum IgG trough levels within the recommended range, a decreased rate of annualized infection, and a low incidence of serious bacterial infections (SBIs). Furthermore, IgPro20 was generally well tolerated with mostly mild or moderate AEs; the most commonly reported AEs were injection site reactions.^9^, ^10^, ^11^, ^12^, ^13^ However, such clinical trials are carefully designed to prove or disprove a null hypothesis, and have limited external validity. It is therefore important that the findings from such studies are verified in a real‐world clinical practice population.

Based on a pivotal Japanese phase 3 clinical study in 24 patients with PID, in which IgPro20 provided similar IgG trough levels to those observed with previous IVIG therapy,^13^ IgPro20 was approved in Japan in 2013 for the treatment of patients with agammaglobulinemia or hypogammaglobulinemia due to PID or SID.^14^ Following its approval, a post‐marketing surveillance (PMS) study was conducted to confirm the safety and effectiveness of IgPro20 during routine clinical use in Japanese patients with hypo‐ or agammaglobulinemia due to PID or SID. Here, we report the findings of this IgPro20 PMS study.

## METHODS

### Study design

This was a multicenter, open‐label, PMS study that evaluated the safety and effectiveness of IgPro20 therapy in patients with agammaglobulinemia or hypogammaglobulinemia due to PID or SID. The surveillance period was from January 2014 to March 2019 and patients were registered between April 2014 and March 2018. Physicians at participating institutions completed a case report form for each registered patient.

The study was conducted at 17 Japanese centers managing patients with hypo‐ or agammaglobulinemia, in accordance with relevant regulations in Japan (Ministerial Ordinance on Good Post‐Marketing Study Practice, Ministry of Health, Labor and Welfare Ordinance Number 171, December 20, 2004). Patients were included in the study if they or a legal guardian gave informed consent. The study protocol was reviewed and approved by the Pharmaceuticals and Medical Devices Agency in Japan prior to study initiation. Ethics committee approval was obtained from each participating hospital, as required; not all participating hospitals required ethics committee approval.

### Patients and treatment

All patients who received IgPro20 for the treatment of hypo‐ or agammaglobulinemia during the study period were eligible for this study, irrespective of whether they were administered treatment at home or in the hospital. Patients participating in the open‐label extension phase of the phase 3 trial “IgPro20_3006” (ClinicalTrials.gov identifier NCT01461018) were also included, if eligible, and continued SCIG therapy.

The IgPro20 treatment schedule, doses, and frequency of administration per week were determined according to the prescribing information, which describes that the usual dosage for subcutaneous injection is 50–200 mg (0.25–1 ml)/kg bodyweight of human IgG once a week. The dosage and frequency of administration per week were adjusted at the discretion of the patient's physician according to each patient's condition. The observation period for each patient was 6 months from the first administration.

### Study objectives, data collection and assessments

Patient data at study registration included demographic and clinical characteristics, including total serum IgG levels prior to IgPro20 administration.

The primary objective of the study was to evaluate the safety of IgPro20 by assessing the number of patients with adverse events (AEs), including serious AEs (SAEs). An AE was defined as any adverse or unintended sign (including abnormal laboratory test values), symptoms, or diseases that occurred when IgPro20 was administered, including the progression or worsening of the primary disease or death. Adverse events that were fatal, life threatening, or resulted in disability, hospitalization or extension of hospitalization, congenital anomaly/birth defect, or any other AEs that were as severe as those mentioned above were defined as serious.

We also evaluated the number of patients with adverse drug reactions (ADRs; i.e., an AE for which a causal relationship with the study drug could not be ruled out) and the incidence of specific ADRs defined as ADRs of special interest (ADRs‐SI). These ADRs were shock/anaphylaxis, aseptic meningitis syndrome, thromboembolism, hepatic dysfunction, acute renal failure, thrombocytopenia, pulmonary edema, hemolysis, and local reactions.

All AEs and ADRs were classified according to the International Council for Harmonization Medical Dictionary for Regulatory Activities, Japanese edition (MedDRA/J) Version 23.0, by system organ class and preferred term.

The effectiveness of IgPro20 was evaluated by comparing the number and type of infections during the 6 months before the first administration of IgPro20 with the number of infections during the observation period. The effectiveness of IgPro20 was also evaluated by the change in serum IgG trough levels, which were measured as a part of general serological tests. For patients who administered SCIG at home, clinical tests (including serology) were performed when they visited the hospital at the physician's discretion. The physicians judged effectiveness based on serum IgG levels during treatment, and the occurrence of infections associated with the primary disease, and other factors.

The mean ± standard deviation (SD) time spent visiting hospital was estimated by the physician based on the number of hospital visits, and the time spent by patients at each hospital visit during treatment with IVIG and with subcutaneous IgPro20.

All AE and effectiveness data, and dosage and administration data, were recorded by participating physicians on the case report during, and on completion of, the 6‐month observation period.

### Statistical analysis

The total number of patients with PID in Japan is approximately 3000,^15^ of whom approximately 800–850 are eligible for immunoglobulin replacement therapy. We estimated that, after the launch of IgPro20, 25 new patients annually, and approximately 200 patients in the next 8 years would receive this SCIG. Therefore, the enrollment period was set as 4 years and the target number of patients for this study was determined to be 100. The safety analysis set included all patients who provided informed consent, received at least one dose of IgPro20, and had a fully completed case report form. The effectiveness analysis set included all patients from the safety analysis set who had at least one post‐baseline effectiveness assessment. Descriptive statistics (number of patients, mean and SD, median and range) were used to summarize continuous effectiveness variables, and frequency and percentage were used to describe categorical safety and effectiveness variables. The correlation between serum IgG level and the weekly IgG dose was analyzed using linear regression analysis.

## RESULTS

### Patients

**FIGURE 1:**
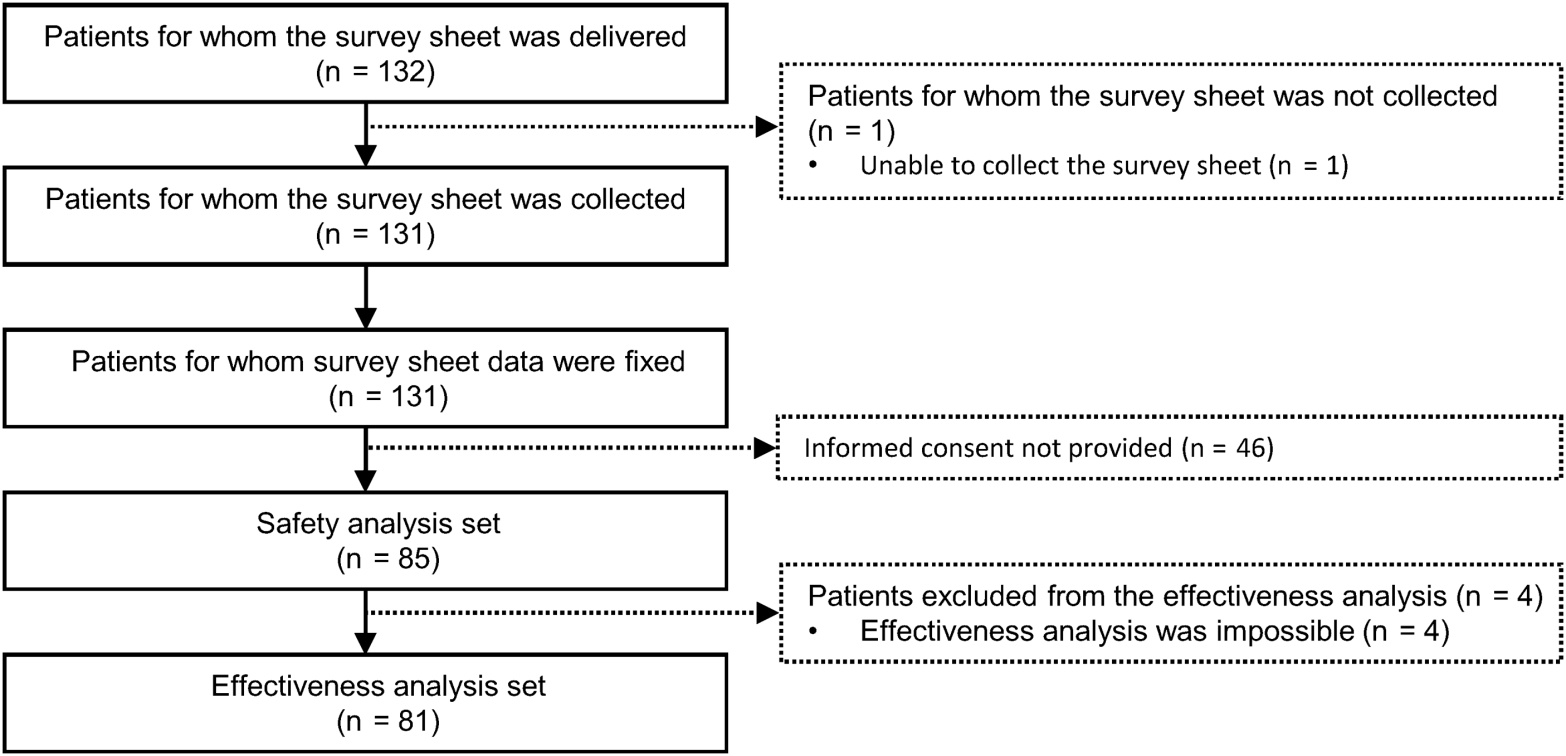
Patient flow

Overall, 132 patients were registered and survey sheet data were available for 131 patients. Informed consent was obtained from 85 patients, who comprised the safety analysis set. The effectiveness analysis included 81 patients because four patients did not have effectiveness data available for analysis (Figure 1).

More than two‐thirds of patients (70.6%) had PID, and 29.4% had SID (Table 1). Of the 74 patients with age data recorded, 22 were infants or children <16 years of age, 12 were aged ≥16 and <19 years, 38 were aged ≥19 years to <65 years and two were aged ≥65 years.

**TABLE 1:** Demographic and patient characteristics of the safety analysis set

Characteristics	Total (*n* = 85)	PID (*n* = 60)	SID (*n* = 25)
Male, *n* (%)	52 (61.2)	40 (66.7)	12 (48.0)
Age, mean ± SD	23.4 ± 15.7 (*n* = 74)	20.3 ± 11.9 (*n* = 49)	29.5 ± 20.1 (*n* = 25)
Age category, *n* (%)			
<1 years	6 (7.1)	4 (6.7)	2 (8.0)
≥1 to <7 years	4 (4.7)	2 (3.3)	2 (8.0)
≥7 to <13 years	5 (5.9)	4 (6.7)	1 (4.0)
≥13 to <16 years	7 (8.2)	6 (10.0)	1 (4.0)
≥16 to <19 years	12 (14.1)	10 (16.7)	2 (8.0)
≥19 to <65 years	38 (44.7)	23 (38.3)	15 (60.0)
≥65 years	2 (2.4)	0	2 (8.0)
Unknown	11 (12.9)	11 (18.3)	0
Height, *n* (%)			
<100 cm	8 (9.4)	4 (6.7)	4 (16.0)
≥100 to <110 cm	3 (3.5)	3 (5.0)	0
≥110 to <120 cm	4 (4.7)	3 (5.0)	1 (4.0)
≥120 to <130 cm	0	0	0
≥130 to <140 cm	2 (2.4)	1 (1.7)	1 (4.0)
≥140 to <150 cm	7 (8.2)	6 (10.0)	1 (4.0)
≥150 to <160 cm	17 (20.0)	9 (15.0)	8 (32.0)
≥160 to <170 cm	27 (31.8)	20 (33.3)	7 (28.0)
≥170 to <180 cm	13 (15.3)	10 (16.7)	3 (12.0)
≥180 to <190 cm	1 (1.2)	1 (1.7)	0
≥190 cm	0	0	0
Unknown	3 (3.5)	3 (5.0)	0
Bodyweight, *n* (%)			
<10 kg	8 (9.4)	4 (6.7)	4 (16.0)
≥10 to <20 kg	5 (5.9)	5 (8.3)	0
≥20 to <30 kg	6 (7.1)	5 (8.3)	1 (4.0)
≥30 to <40 kg	6 (7.1)	4 (6.7)	2 (8.0)
≥40 to <50 kg	20 (23.5)	12 (20.0)	8 (32.0)
≥50 to <60 kg	19 (22.4)	15 (25.0)	4 (16.0)
≥60 to <70 kg	12 (14.1)	7 (11.7)	5 (20.0)
≥70 to <80 kg	4 (4.7)	4 (6.7)	0
≥80 to <90 kg	1 (1.2)	1 (1.7)	0
≥90 to <100 kg	1 (1.2)	1 (1.7)	0
≥100 kg	1 (1.2)	0	1 (4.0)
Unknown	2 (2.4)	2 (3.3)	0
Initial serum IgG concentration, *n* (%)			
<400 mg/dl	20 (23.5)	12 (20.0)	8 (32.0)
≥400 to <700 mg/dl	30 (35.3)	15 (25.0)	15 (60.0)
≥700 to <1000 mg/dl	21 (24.7)	19 (31.7)	2 (8.0)
≥1000 mg/dl	5 (5.9)	5 (8.3)	0
Unknown	9 (10.6)	9 (15.0)	0
Primary disease, *n* (%)			
PID	60 (70.6)	60 (100.0)	–
CVID	30 (35.3)	30 (50.0)	–
XLA	15 (17.7)	15 (25.0)	–
ADA2 deficiency	3 (3.5)	3 (5.0)	–
Others	12 (14.1)	12 (20.3)	–
SID	25 (29.4)	–	25 (100.0)
Nephrotic syndrome	6 (7.1)	–	6 (24.0)
Post‐lung transplant	4 (4.7)	–	4 (16.0)
PLGE	2 (2.4)	–	2 (8.0)
Others	13 (15.3)	–	13 (52.0)
Post‐immunosuppressive therapy	11 (12.9)	–	11 (44.0)
Previous IVIG treatment, *n* (%)	56 (65.9)	40 (66.7)	16 (64.0)
ADRs with previous IVIG treatment	9 (16.1)	8 (13.3)	1 (4.0)
Total IgPro20 administration period, *n* (%)			
<4 weeks	9 (10.6)	3 (5.0)	6 (24.0)
≥4 to <12 weeks	6 (7.1)	3 (5.0)	3 (12.0)
≥12 to <24 weeks	6 (7.1)	3 (5.0)	3 (12.0)
≥24 weeks	64 (75.3)	51 (85.0)	13 (52.0)

Abbreviations: ADA2, adenosine deaminase 2; ADR, adverse drug reactions; CVID, common variable immunodeficiency; IgG, immunoglobulin G; IVIG, intravenous immunoglobulin; PID, primary immunodeficiency syndrome; PLGE, protein‐losing gastroenteropathy; SID, secondary immunodeficiency syndrome; XLA, X‐linked agammaglobulinemia.

In the PID subgroup, the most common type of PID was CVID (50.0% of PID patients), followed by XLA (25.0%), and adenosine deaminase 2 deficiency (5.0%; Table 1). One patient (1.7%) each had severe combined immunodeficiency, X‐linked inhibitor of apoptosis deficiency, combined immunodeficiency, cartilage‐hair hypoplasia syndrome, ataxia telangiectasia, activated PI3K delta syndrome, hyper‐IgM syndrome, IgG2 deficiency, Kabuki syndrome, or were under investigation for their disease type.

In the SID subgroup, the most common primary diseases were nephrotic syndrome (24.0% of SID patients), post‐lung transplant (16.0%), and PLGE (8.0%; Table 1). Other SIDs included end‐stage renal disease, post‐hematopoietic stem cell transplant, and post‐immunosuppressive therapy. None of the patients with SID had a malignancy.

Overall, 56 patients (65.9%) had been treated with IVIG previously, 40 in the PID subgroup and 16 in the SID subgroup. Nine of 56 patients (16.1%) had experienced an ADR during the previous IVIG treatment, including eight patients in the PID subgroup and one in the SID subgroup (Table 1).

### Treatments

**FIGURE 2:**
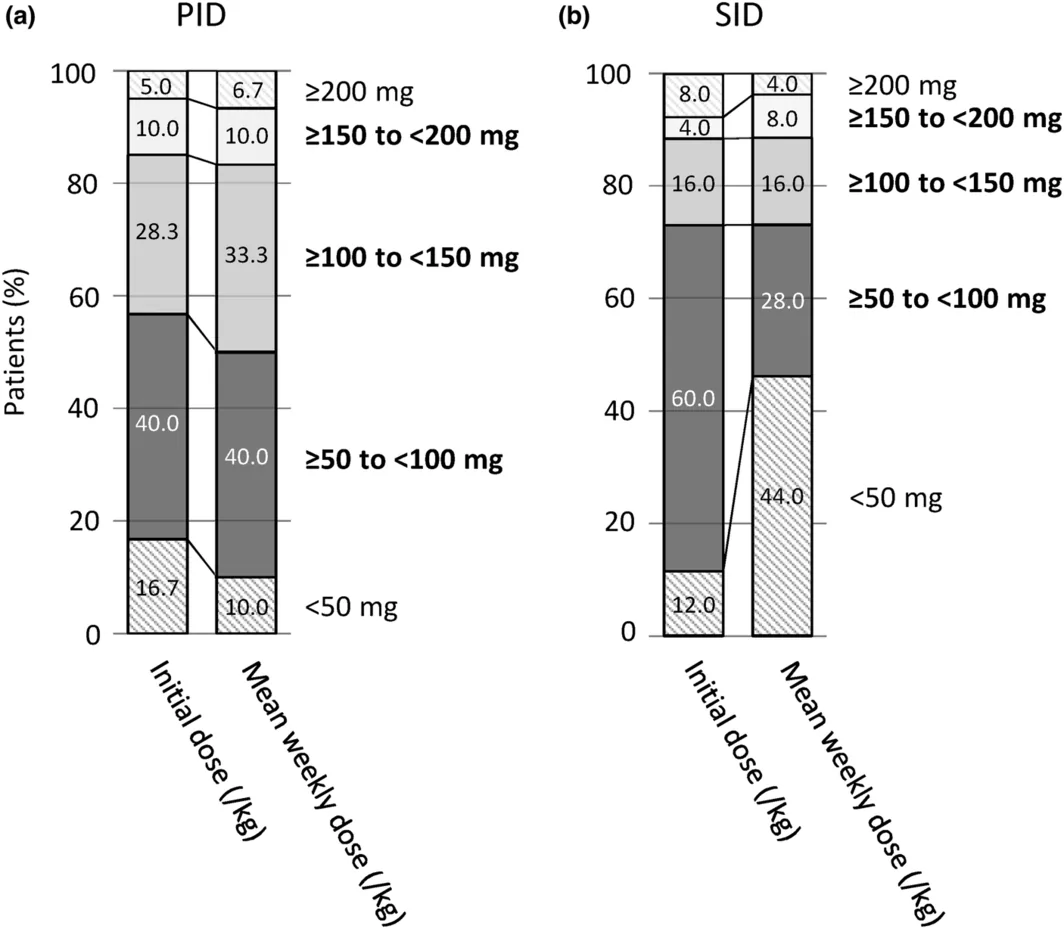
IgPro20 dosage and administration status (a) in patients with primary immunodeficiencies (PID) and (b) in patients with secondary immunodeficiencies (SID)

IgPro20 dose information was available for 85 patients at the start of treatment and for 78 patients during treatment. Approximately 80% of patients (67/85) started IgPro20 at a dose of 50–200 mg (0.25–1 ml)/kg bodyweight as instructed in a package insert (Figure 2). During the observation period, 30 patients (35.3%) overall were administered IgPro20 once a week as recommended. In the PID subgroup, the starting dose of IgPro20 was the usual recommended dose of 50–200 mg (0.25–1 ml)/kg in 47/60 patients (78.3%) and the mean weekly dose during treatment was 50–200 mg (0.25–1 ml)/kg bodyweight of human IgG once a week in 50/60 patients (83.3%). In contrast, in the SID subgroup, 20/25 patients (80.0%) received the usual dose at the start of treatment, and the mean weekly dose during treatment was 50–200 mg (0.25–1 ml)/kg bodyweight of human IgG once a week in 13/25 patients (52.0%).

The overall percentage of patients treated with IgPro20 for 24 weeks was 75.3%. Of these, 79.7% were in the PID subgroup and 20.3% were in the SID. Eleven patients (PID: *n* = 4; SID: *n* = 7) discontinued IgPro20 during the observation period. The reasons for treatment discontinuation were AEs – PID: *n* = 2 (injection site pain; *n* = 1, aseptic meningitis syndromes; *n* = 1); SID: *n* = 3 (aseptic meningitis syndromes; *n* = 1, hives; *n* = 1, bronchiolitis obliterans syndrome; *n* = 1); patients changed to a different hospital (PID: *n* = 1; SID: *n* = 1); patient's request (PID: *n* = 1), and others, including increased IgG levels associated with treatment of the underlying disease (SID: *n* = 3). None of the patients stopped IgPro20 treatment because of lack of effectiveness.

### Safety

Adverse events were observed in 39 patients (45.9%) overall; 28 patients in the PID subgroup (46.7%) and 11 patients in the SID subgroup (44.0%) reported AEs. In total, 27 patients (31.8%) developed at least one ADR with onset during the observation period – 21 in the PID subgroup and six in the SID subgroup. The most common ADR was injection site reaction, which developed in 17 patients (20.0%) overall (Table 2).

**TABLE 2:** Adverse drug reactions in the safety analysis set

ADR^a^, *n* (%)	Total (*n* = 85)	PID (*n* = 60)	SID (*n* = 25)
At least one ADR	27 (31.8)	21 (35.0)	6 (24.0)
Infections	2 (2.4)	1 (1.7)	1 (4.0)
Aseptic meningitis syndromes^b^	2 (2.4)	1 (1.7)	1 (4.0)
Neurological disorders	3 (3.5)	2 (3.3)	1 (4.0)
Headache	3 (3.5)	2 (3.3)	1 (4.0)
Hypoesthesia	1 (1.2)	0	1 (4.0)
Gastrointestinal disorders	3 (3.5)	1 (1.7)	2 (8.0)
Diarrhea	1 (1.2)	1 (1.7)	0
Nausea	2 (2.4)	0	2 (8.0)
Vomiting	1 (1.2)	0	1 (4.0)
Skin and subcutaneous tissue disorders	4 (4.7)	2 (3.3)	2 (8.0)
Acne	1 (1.2)	0	1 (4.0)
Prurigo	1 (1.2)	1 (1.7)	0
Exanthema	1 (1.2)	0	1 (4.0)
Hives	2 (2.4)	1 (1.7)	1 (4.0)
Musculoskeletal and connective tissue disorders	1 (1.2)	0	1 (4.0)
Musculoskeletal pain	1 (1.2)	0	1 (4.0)
Congenital, familial and genetic disorders	1 (1.2)	1 (1.7)	0
Congenital anomaly	1 (1.2)	1 (1.7)	0
Injection site reactions	17 (20.0)	16 (26.2)	1 (4.0)
Induration	1 (1.2)	0	1 (4.0)
Pain	5 (5.9)	5 (8.3)	0
Erythema	14 (16.5)	14 (23.3)	0
Pruritus	1 (1.2)	1 (1.7)	0
Swelling	6 (7.1)	6 (10.0)	0
General disorders	4 (4.7)	3 (5.0)	1 (4.0)
Chest discomfort	1 (1.2)	1 (1.7)	0
Chest pain	1 (1.2)	1 (1.7)	0
Peripheral edema	1 (1.2)	0	1 (4.0)
Fever	1 (1.2)	1 (1.7)	0

^**a**^ ADRs were coded using MedDRA/J version 23.0.

^b^ ADRs‐SI.

Abbreviations: ADR, adverse drug reaction; ADRs‐SI, adverse drug reactions of special interest; PID, primary immunodeficiency syndrome; SID, secondary immunodeficiency syndrome.

In total, SAEs were recorded in four patients, three in the PID subgroup (5.0**%**) and one in the SID subgroup (4.0%). One patient with PID died of a congenital anomaly (date of death unknown); the death was considered to be unrelated to IgPro20 treatment.

Among the nine patients who had experienced an ADR during their previous IVIG treatment, three experienced the same ADR in this study.

Adverse drug reactions of special interest occurred in 19 patients overall; 17 (28.3%) in the PID subgroup and two (8.0%) in the SID subgroup (Table 3). Aside from injection site reactions (20.0%) and aseptic meningitis syndrome (2.4%), no other ADRs‐SI were observed (Table 3).

**TABLE 3:** Adverse drug reactions of special interest in the safety analysis set

	Total (*n*)	ADRs‐SI, *n* (%)	Injection site reactions, *n* (%)	Aseptic meningitis, *n* (%)
Total	85	19 (22.4)	17 (20.0)	2 (2.4)
Age				
<15 years	19	9 (47.4)	8 (42.1)	1 (5.3)
≥15 to <65 years	53	10 (18.9)	9 (17.0)	1 (1.9)
≥65 years	2	0	0	0
Unknown	11	0	0	0
Bodyweight				
<45 kg	32	10 (31.3)	8 (25.0)	2 (6.3)
≥45 to <65 kg	43	7 (16.3)	7 (16.3)	0
≥65 kg	8	2 (25.0)	2 (25.0)	0
Unknown	2	0	0	0
Primary disease				
PID	60	17 (28.3)	16 (26.7)	1 (1.7)
SID	25	2 (8.0)	1 (4.0)	1 (4.0)
Other	6	0	0	0

Abbreviations: ADR, adverse drug reaction; ADRs‐SI, adverse drug reactions of special interest; PID, primary immunodeficiency syndrome; SID, secondary immunodeficiency syndrome.

### Effectiveness

#### Infections

In the 6 months prior to IgPro20 initiation, 41 infections developed in 27 patients (0.54 infections per patient; Table 4); 30 developed in patients with PID (0.57 infections per patient) and 11 developed in patients with SID (0.48 infections per patient). Overall, 29 patients had 33 infections during the 6 month observation period after initiating treatment with IgPro20 (0.39 infections per patient): there were 27 infections in 23 patients in the PID subgroup (0.46 infections per patient) and six infections in six patients in the SID subgroup (0.23 infections per patient; Table 4). The most common type of infection was repeated respiratory tract infection (including otitis media and sinusitis), which occurred in 19 patients during the observation period (17 with PID and two with SID; Table 4). Serious bacterial infections were observed in six patients prior to starting IgPro20 (two in the PID subgroup and four in the SID subgroup). During the observation period, one patient in the SID subgroup had an SBI, and this was one of the patients who had an SBI in the baseline period (Table 4). The overall rate of SBI during IgPro20 treatment was 7.9%.

**TABLE 4:** Incidence of infection

	Total		PID subgroup		SID subgroup	
	Before^a^ (*n* = 76)	After^b^ (*n* = 85)	Before^a^ (*n* = 53)	After^b^ (*n* = 60)	Before^a^ (*n* = 23)	After^b^ (*n* = 25)
Any infections						
No. (%) of patients	27 (35.5)	29 (34.1)	19 (35.8)	23 (38.3)	8 (34.8)	6 (24.0)
No. of infections	41	33	30	27	11	6
No. of infections per patient	0.54	0.39	0.57	0.45	0.48	0.24
Specific infections, no. (%) of patients						
Repeated RTI^c^	16 (21.1)	19 (22.4)	14 (26.4)	17 (28.3)	2 (8.7)	2 (8.0)
SBI	6 (7.9)	1 (1.2)	2 (3.8)	0	4 (17.4)	1 (4.0)
Bronchiectasis	5 (6.6)	1 (1.2)	5 (9.4)	1 (1.7)	0	0
Pyoderma	2 (2.6)	1 (1.2)	2 (3.8)	1 (1.7)	0	0
Prolonged diarrhea	1 (1.3)	0	1 (1.9)	0	0	0
Oral candidiasis	2 (2.6)	0	0	0	2 (8.7)	0
Other infections	9 (11.8)	11 (12.9)	6 (11.3)	8 (13.3)	3 (13.0)	3 (12.0)

^a^ Before = infection rate during the 6 months before IgPro20 administration.

^b^ After = infection rate during the 6 months after initiating treatment with IgPro20.

^c^ Including otitis media and sinusitis.

Abbreviations: PID, primary immunodeficiency syndrome; RTI, respiratory tract infection; SBI, serious bacterial infection; SID, secondary immunodeficiency syndrome.

#### Serum IgG trough level

In IVIG‐naïve patients (*n* = 17), the mean ± SD IgG trough level was 585 ± 380 mg/dl at baseline and 842 ± 212 mg/dl after treatment with IgPro20. Patients who had previously received treatment with IVIG (*n* = 27) had a mean ± SD IgG trough level of 697 ± 243 mg/dl at baseline and 862 ± 233 mg/dl after treatment with IgPro20.

**FIGURE 3:**
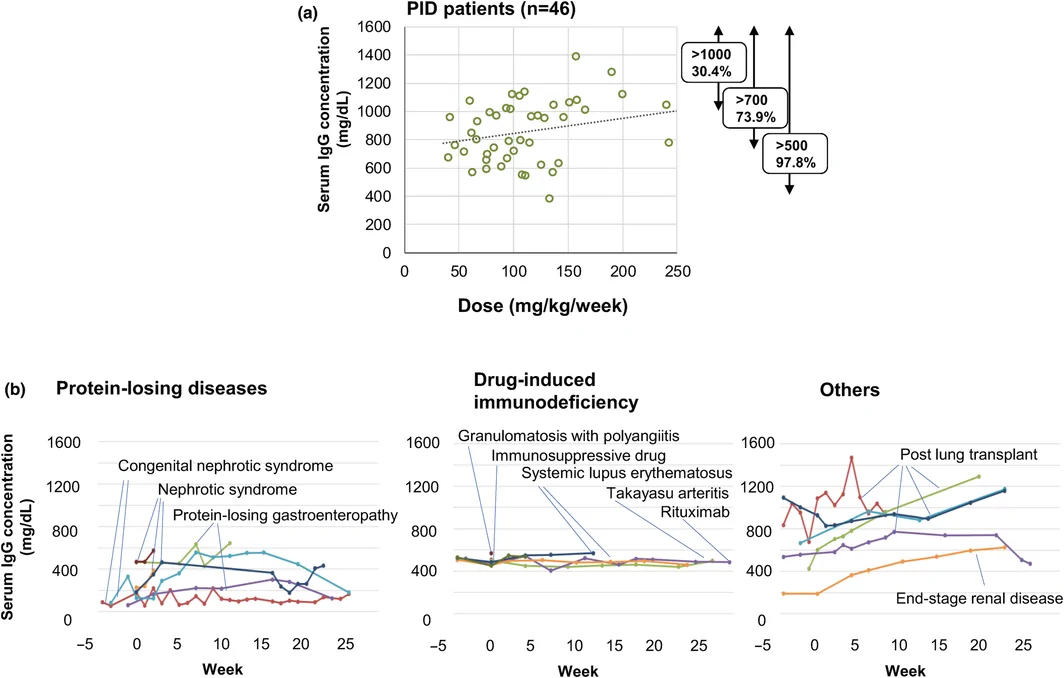
Dose and serum immunoglobulin G (IgG) trough levels after IgPro20 administration; (a) serum IgG levels against IgPro20 dose in patients with primary immunodeficiencies (PID) after IgPro20 administration and (b) time course of serum IgG levels of 21 patients with secondary immunodeficiencies (SID)

Figure 3a shows the IgG trough levels (mg/dl) in 46 PID patients after IgPro20 SCIG treatment plotted against dose of administration (mg/dl/week). No correlation was found between individual IgG trough level and the weekly IgG dose (Figure 3a). IgG trough levels after IgPro20 treatment were higher than 500 mg/dl in 45/46 patients (97.8%), higher than 700 mg/dL in 34 patients (73.9%), and higher than 1000 mg/dl in 14 patients (30.4%; Figure 3a).

Figure 3b shows the time course of serum IgG concentrations after IgPro20 in 21 patients in the SID subgroup; these time courses showed different patterns according to the underlying primary disease and treatments.

#### Physician‐judged effectiveness

Treatment was considered effective by physicians in all 81 patients comprising the effectiveness analysis set. In the 56 patients who had been treated with IVIG previously, the mean ± SD time spent visiting hospital was 234 ± 285 min during the 6‐month period before treatment and 125 ± 81 min during the 6 month observation period after initiating IgPro20 treatment.

## DISCUSSION

Subcutaneous immunoglobulin offers numerous advantages for patients and caregivers, principally because of the ability to self‐administer the medication at home. Most of the patients in our study had been previously receiving IVIG, and the switch to SCIG substantially reduced the amount of time patients spent visiting the hospital. International studies indicate that patients have better quality of life and prefer treatment at home with SCIG compared with hospital‐based IVIG.^16^ Moreover, switching from IVIG to home‐based SCIG can result in substantial cost savings for healthcare payers.^17^, ^18^, ^19^, ^20^

This study, the largest to date with IgPro20 in patients with agammaglobulinemia or hypogammaglobulinemia due to either PID or SID, showed that this SCIG was well tolerated and safe, and was considered effective by the treating physicians, when used in real‐world clinical practice in Japan. The analysis of serum IgG trough levels also showed an acceptable increase in IgG level, and patients spent less time visiting the hospital during IgPro20 treatment than they did before starting this treatment.

The approved indications for IgPro20 in Japan differ from those in other countries. For example, in the US, IgPro20 is only approved for the treatment of PIDs,^21^ and in Europe, it is approved for some SIDs, such as MM,^22^ whereas, in Japan, it is approved for the treatment of PIDs and SIDs presenting with hypogammaglobulinemia, including drug‐induced, protein‐losing diseases, and disease‐related antibody deficiency.

The most common ADRs observed in our study were injection site reactions, which was one of the ADRs‐SIs. This is consistent with previous clinical trials in patients with PID.^9^, ^10^, ^11^, ^12^, ^13^ All ADRs, except two cases of gastroenteritis, were confirmed causal. All of the causal ADRs reported in this study have been reported in previous studies. Among the ADRs‐SI, only local injection site reaction (*n* = 17; 20%) and aseptic meningitis syndrome (*n* = 2; 2.4%) were seen in our study. The incidence of local reactions was considerably lower than that observed in previous pivotal studies (49–100%).^9^, ^10^, ^13^ This may be because local reactions reportedly occurred early in the treatment period in previous studies, but the current study includes patients who received IgPro20 over a longer observation period than that of previous studies. In addition, 44.0% of patients with SID were receiving concomitant immunosuppressive drugs, which may have reduced the risk of local reactions. Two cases of aseptic meningitis occurred in patients with PID and SID, at 3 and 19 days after IgPro20 administration, respectively. This disorder has previously been reported in patients treated with IVIG and is related to immune reactions between therapeutic and endogenous IgG.^23^, ^24^, ^25^ The incidence of aseptic meningitis following IVIG administration has previously been reported as 0.6% in one study (*n* = 1324)^26^ and 1.0% in another study (*n* = 384).^27^ Although this incidence is slightly lower than that reported after IgPro20 administration in this study (2.4%), the patient population of the current study (*n* = 85) was considerably smaller than those of the previous studies,^26^, ^27^ making comparisons between studies difficult. Nevertheless, when treating patients with IgG, physicians should monitor patients for signs that may indicate aseptic meningitis syndrome, such as neurologic changes or deterioration, headache, fever, and vomiting.^28^

Consistent with previous pivotal clinical trials in Japan,^13^ the US,^9^ and Europe,^10^ none of the patients with PID in the current observational study developed an SBI. One patient with SID (after a lung transplant) had an SBI during treatment with IgPro20 in the current study; this patient had also experienced an SBI during the 6 months before starting IgPro20. This patient's trough serum IgG level was 826.0 mg/dl, which may have been the result of an inadequate IgPro20 dose. However, the incidence of SBIs in patients with SID decreased from 17.4% (before IgPro20) to 3.9% (after IgPro20) and, in the total population, the SBI incidence decreased from 7.9% to 1.2% during IgGPro20 treatment. The infection rate per patient decreased from 0.54 to 0.39 overall, with the most marked change occurring in the subgroup with SID (from 0.48 infections per patient in the 6 months before IgPro20 to 0.12 infections per patient during IgPro20).

In this study, the effectiveness of IgPro20 was also confirmed for the treatment of patients with SID. For SID patients, there has been only one prospective randomized trial with IgPro20 in MM patients with hypogammaglobulinemia (*n* = 46).^29^ In this study, IgPro20 significantly (all *P* < 0.001) reduced the total number of infections, serious infections, and respiratory tract infections, compared with no IgG treatment. In the future, randomized controlled trials and large scale prospective clinical studies are needed for other SIDs.

Serum IgG trough levels are recommended to be ≥1000 mg/dl to prevent pneumonia, according to one meta‐analysis in patients with PID.^30^ In our study, 97.8% of patients in the PID subgroup achieved serum IgG trough levels of >500 mg/dl, but only 30% had a trough level of >1000 mg/dl. Nonetheless, no infections were observed. This may be because the IgG trough levels required to prevent infection varies between individual patients, a concept known as biologic IgG level.^31^ The levels and time courses of serum IgG levels in patients in the SID subgroup were different depending on the underlying disease. Notably, the patients with drug‐induced immunodeficiency achieved serum IgG trough levels of >500 mg/dl only temporarily, and none of them achieved a serum IgG trough level of >600 mg/dl. However, no infections were found in these patients. In the SID subgroup, the serum IgG trough level was different for each primary disease; therefore, it may be necessary to determine the target IgG trough level for each primary disease in patients with SID in the future.

Our study has some limitations. First, it was an observational study, with no control group. Second, our study had an observation period of 24 weeks, whereas patients with PID and SID often require longer term IgG replacement therapy. Future research should include randomized controlled trials, and studies investigating the long‐term safety and effectiveness of IgPro20 in patients with agammaglobulinemia and hypogammaglobulinemia due to PID or SID.^12^

### CONCLUSIONS

This study is the largest to date in patients receiving SCIG IgPro20. During the 6 month observation period of this PMS study of IgPro20 in a real‐world clinical setting in Japan, no safety concerns were observed, and the effectiveness in patients with agammaglobulinemia or hypogammaglobulinemia due to both PID and SID was confirmed.

## AUTHOR CONTRIBUTIONS

Tetsushi Akasaki and Hideo Usui contributed to conception and design of the study; Taku Fukushima, Masayuki Matsumaru, Tetsushi Akasaki and Hideo Usui contributed to acquisition and analysis of the data; Kohsuke Imai, Tomonori Ishii, Shigeaki Nonoyama, Takahiro Yasumi, Hirokazu Kanegane, Taku Fukushima, Masayuki Matsumaru, Tetsushi Akasaki and Hideo Usui contributed to interpretation of the data and writing (drafting and revising) of the manuscript. All of the authors read and approved the final manuscript for publication.

## CONFLICT OF INTEREST

Kohsuke Imai received support from CSL Behring K.K. for development of the present manuscript. Tomonori Ishii has participated on speaker bureaus for GlaxoSmithKline, Chugai Pharmaceutical Co., Ltd., Janssen Pharmaceutical K.K., Astellas Pharma Inc., and Asahi Kasei Pharma Corporation. Taku Fukushima, Masayuki Matsumaru, Tetsushi Akasaki and Hideo Usui are employees of CSL Behring. K.K. Shigeaki Nonoyama, Takahiro Yasumi and Hirokazu Kanegane declare no conflicts of interest.
